# “Something to Live for”: Experiences, Resources, and Personal Strengths in Late Adulthood

**DOI:** 10.3389/fpsyg.2019.02452

**Published:** 2019-10-31

**Authors:** Pninit Russo-Netzer, Hadassah Littman-Ovadia

**Affiliations:** ^1^Department of Counseling and Human Development, University of Haifa, Haifa, Israel; ^2^Department of Behavioral Sciences and Psychology, Ariel University, Ariel, Israel

**Keywords:** aging, adulthood, well-being, character strengths, existential concerns, qualitative methodology

## Abstract

Due to increased life expectancy, the population segment of older adults has grown the fastest. The global phenomenon of population aging raises important questions regarding successful, positive, active, and meaningful aging. Given that aging is often characterized by declines in physical and mental health and increased risk for social isolation and depression, and given that the concept of well-being in old age is both elusive and complex, the present study explored how aging is experienced through a “bottom-up,” open-ended approach. Thirty-one in-depth semi-structured personal interviews were conducted with adults aged 60 and above in order to explore the question: what concerns older adults in their day-to-day living, and what are their perceived resources? The findings illuminated three prominent themes: (1) central concerns described by the participants as characterizing their experience at this life stage; (2) strategies employed by the participants to cope with concerns and to live a meaningful life in old age; and (3) resources and character strengths that facilitate coping strategies and enable thriving. Theoretical and practical implications of the findings are discussed.

## Introduction

Advances in medicine and medical technology have led to a continuous rise in life expectancy. Due to the expansion of the lifespan, the population segment of older adults grows fastest. Worldwide, the proportion of people age 60 and above is growing faster than any other age group. In 2025, there will be a total of 1.2 billion, and by 2050, there will be about 2 billion people over the age of 60 [WHO (World Health Organization), 2002]. This global phenomenon of population aging raises important questions regarding aging well. In the last decades, aging well was conceptualized by different theoretical frameworks as active aging [WHO (World Health Organization), 2011], as well as healthy, positive, optimal successful, and meaningful aging. These different indicators of positive aging highlight different indicators of positive psychological functioning such as quality of life, social engagement, meaning in life, positive emotions, and life satisfaction. The purpose of this qualitative study was to explore older adults’ own experiences of their actual aging, their perceived resources that enable them to cope with their concerns and challenges in their day-to-day living.

### Challenges and Well-Being in Old Age

The aging process comprises a key transitional phase in one’s life, at which age-related challenges and the relative proximity of death (Erikson, 1968) involves recurrent experiences of loss and decline in central aspects of life, such as family, friends, life transitions, capacities, and physical condition (e.g., Greenglass et al., 2006; Sundström et al., 2018). These experiences press existential issues, such as fear of death, existential vacuum, and social isolation (e.g., Turner and Lloyd, 1999; Cicirelli, 2006), and contribute to an overall sense of loneliness (Graneheim and Lundman, 2010).

Alongside the recognition of aging as a trajectory of decline, other approaches within the social sciences, such as humanism (Maslow, 1954), logotherapy (Frankl, 1959), and positive psychology (e.g., Seligman and Csikszentmihalyi, 2000), paved the way toward a scientific interest in the study of optimal and even positive development in aging. These approaches have shown that despite age-related increase in losses and physical and mental challenges, negative effect does not increase with age, but rather remains stable (e.g., Diener and Suh, 1998; Mroczek and Kolarz, 1998) or even decreases (e.g., Charles et al., 2001; Hudson et al., 2016), and older individuals seem to be able to produce more positive life stories and reviews, when engaging with their past (Lieblich, 2014).

In her model, Ryff (1995) suggested six pillars of psychological well-being in adult life, including self-acceptance, purpose in life, environmental mastery, positive relationships, autonomy, and personal growth. In congruent with this well-researched model, aging well has been conceptualized using various theoretical frameworks, such as healthy aging, positive aging, active aging, and successful aging (e.g., Poon and Cohen-Mansfield, 2011; Foster and Walker, 2014; Martin et al., 2015). However, these conceptualizations, terms, and measures are inconsistent, so much so that the meaning of aging-well is often more implied than delineated, present limitations to research on aging-well. Rowe and Kahn (1997) were among the first to characterize the prosperous lifestyle and worthy living in old age, highlighting three main elements of successful aging: health, high cognitive and physical functioning, and a fruitful and positive social circle. However, Rowe’s and Kahn’s paradigm neglected the consideration of what aging *means* to older adults, a limitation that can be addressed through qualitative discovery and self-report studies. Indeed, when older adults were asked to describe their own experiences, a discrepancy appeared between the *objective criteria* (disability and disease) and older individuals’ *subjective experiences* and *definitions* (of unsuccessful aging or successful aging) (Montross et al., 2006). Similar findings emerged in other qualitative studies on successful aging (Knight and Ricciardelli, 2003; Reichstadt et al., 2007; Lieblich, 2014). Accordingly, the WHO (World Health Organization) (2002), in their policy framework, defined active aging as “the process of optimizing opportunities for health, participation and security in order to enhance quality of life as people age” (p. 12).

The abovementioned theoretical frameworks yielded studies, which identified concerns faced by older people, showing how specific challenges relate to aging well. However, a round and holistic examination of concerns and challenges experienced in old age is missing, including the ways and personal resources with which these challenges are addressed.

### Resources in Old Age

The integration of positive psychology and the development in the study of positive aging, alongside more traditional research in the field, is becoming more probable with the advancement of medical care and generally higher quality of life (Lieblich, 2014). The focus on improving current functioning and increasing well-being affords renewed in-depth impetus to research on potential resources in aging. For example, the importance of meaning in life to successful aging has been established through ample evidence, including the domains of physical health and longevity (e.g., Boyle et al., 2009; Ryff et al., 2016), as well as in improved mental health outcomes (Musich et al., 2018). Given that life meaning has significance in how aging people make sense of their lives, particularly as a factor in successful aging, more knowledge can be derived from older adults’ own accounts of their experiences, an approach employed in previous studies (e.g. Lieblich, 2014), providing vivid insight into the construction of meaning in the participants’ experiences. Studies exploring positive affect in aging, however, show inconsistent findings, with some indicating increase in positive emotions with age (Gross et al., 1997; Mroczek and Kolarz, 1998), while others stability (Gross et al., 1997; Kunzmann et al., 2013) or their decline with age (Diener and Suh, 1998; Hudson et al., 2016). Another growing area of research in positive psychology relevant to aging is strengths, and specifically character strengths. Defined as durable individual traits, manifested in thought, feeling, and behavior in various degrees in different people (Peterson and Seligman, 2004), character strengths can be used (and understood) as personal resources in dealing with concerns in old age.

Various studies demonstrate the importance of strengths endorsement and deployment to a host of positive psychological outcomes, such as life satisfaction and positive affect (Littman-Ovadia and Lavy, 2012), self-acceptance, a sense of purpose in life, environmental mastery, physical and mental health (Leontopoulou and Triliva, 2012), coping with daily stress (Brooks, Unpublished), and resilience to stress and trauma (e.g., Park and Peterson, 2009). In one of the first studies to examine strengths and their contribution to well-being in different age groups, hope, citizenship, and loving relationships were found as predictors of life satisfaction in older adults, aged 60 and above (Isaacowitz et al., 2003). Encouraging results regarding the potential to acquire and cultivate strengths at old age were reported in Owens et al. (2018) study which examined strength development from a lifespan perspective, and found that although people’s most dominant strengths are stable throughout life, strengths can fluctuate throughout the lifespan. However, most studies conducted within the field of positive psychology have not paid sufficient direct attention to aging population (Araújo et al., 2017; Greenawalt et al., 2019), while most of them were limited to “top-down” quantitative designs. Along these lines, Battersby and Phillips (2016), advocated the importance of qualitative methods to explore “what is meaningful for older adults,” as this construct “is not well understood” (p. 199).

In sum, aging has been viewed as multidimensional, involving various aspects, such as chronological, biological, social, cultural, spiritual, and psychological (e.g., Moyle et al., 2014). Given that aging is a varied and multifaceted experience that depends on a multitude of factors, recent calls emphasize that the definition of successful aging must also take into account what the individuals themselves value, rather than rely on objective measures of physical health, to enable a more holistic bio-psycho-social view (e.g., Martin et al., 2015). Furthermore, given that culture plays an important role in individuals’ values, assumptions, and needs (Markus and Kitayama, 1991), the phenomenon of aging and the experience of its varied potential challenges and resources may have different meanings, manifestations and levels of salience across diverse range of cultures and societies.

### Older Adults in Israel

The Israeli society, where the present study was conducted, introduces a few unique cultural characteristics which include, for example, the prominence of existential threats, a sense of collective vulnerability, uncertainty, and insecurity, all coupled with dialectic worldviews as part of a multicultural immigrant society (e.g., Ezrachi, 2004). In 2016, Israel’s population of adults aged 60 and older reached 15.6%, and is expected to keep rising in the coming years (CBS, 2018). The majority of the aging population is composed of immigrants, who had to flee the Holocaust in Europe, persecution in North Africa and the Middle East, or emigrated from the former Soviet Union after its dissolution. In addition, many older Israeli adults experience a blend of traumatic events, such as exposure to warfare (e.g., Solomon and Ginzburg, 1998; Palgi et al., 2015). Findings from SHARE-Israel (Survey of Health, Aging and Retirement in Europe) suggest that more than 75% of Israelis aged 50 or older reported to have experienced at least one traumatic event (Shmotkin and Litwin, 2009), and that Israeli adults are more depressed than European counterparts (Litwin, 2009). Against this background, the current study sought to complement previous knowledge in the field through a “bottom-up,” open-ended exploration of Israeli older adults’ own perspectives regarding the experience and potential resources at this life stage.

## Materials and Methods

This study adopted a qualitative-phenomenological prism, due to its focus on exploring the meaning of phenomena in human experience (Giorgi, 1997) from the perspectives of the individuals themselves (Maykut and Morehouse, 1994). Phenomenology is essentially a qualitative inquiry into humans’ “lived experience” (van Manen, 1990) through the exploration of people’s actions, relations, and situations. Phenomenology thus attempts to identify the internal meaning structures or essences of lived experience through a study of its particulars (van Manen, 1990).

### Participants

Participants were recruited using a variety of methods to obtain as broad and diverse a perspective of the phenomenon as possible, such as online advertisements and direct recruitment by research assistants, who solicited participants at established community venues for the elderly, such as day centers and community centers. In addition, participants were recruited via the snowball technique (i.e., in which one participant recommends other potential interviewees; Babbie, 1995). The open and broad invitation read, “For a study that focuses on the experience of life in late adulthood, we are seeking participants interested in sharing their experiences in an interview of 1.5–2 h.” Those responding to this invitation were contacted by phone, provided with an explanation of the study, and assured of confidentiality and anonymity.

The sample size in the present study was determined by the saturation principle, as is customary in qualitative studies: Data were collected and analyzed until no new themes emerged (Padgett, 1998). The sample comprised the first 31 people who responded to this invitation and met the criterion of being at least 60 years of age. The participants were all Israeli Jews, 17 males and 14 females. Ages ranged between 60 and 83 years (*M*_age_ = 70.87, SD_age_ = 6.24). Participants’ level of education ranged from high school (35.4%) to higher education (64.4%). Most participants (77.4%) were married, 9.6% divorced, and 12.9% were widows or widowers.

### Procedure

The study received ethical approval by the IRB in the first author’s university. In-depth, face-to-face, semi-structured interviews were employed, each lasting between 1.5 and 2 h. The interviews were audiotaped and later transcribed verbatim. Prior to each interview, the participants signed an informed consent form, which specified the purpose of the research, its procedures, and the voluntary nature of participation, as well as the right to stop participating in the research at any time and issues of confidentiality. The interviews were conducted by a team of three research assistants. Prior to the interviews, the interviewers were trained in the preparation and conduct of the semi-structured interviews to be used (Spradley, 1979), receiving feedback from the authors regarding pilot interviews they conducted. The interview protocol was designed to allow focus without imposing a specific sequence of questions, and included several main content categories: the participants’ aging experience, their perceived challenges and resources in their day-to-day living.

All the interviews began with background information questions, followed by a general, open-ended question inviting the participants to speak about how they experience their lives today at this life stage, allowing them to share spontaneously and describe their personal and subjective experience as freely as possible in their own words. For example, interviewees were asked “How do you experience this stage of your life?” and “How would you describe it?”. The participants were asked additional open-ended probing questions to encourage them, when necessary, to elaborate, clarify meanings, reveal unexplored issues, or provide further details and examples to attain an in-depth understanding of their subjective experiences (van Manen, 1990). For example, the participants were asked to further describe specific experiences or situations they mentioned by posing follow-up questions, like, “You spoke about ‘such and such’; can you tell me more about that?” (Giorgi and Giorgi, 2003); or “What was it like?”; “can you give me an example?”, and “What helps you cope with what you just mentioned?”; “Can you describe that particular experience/incident in more detail?” (van Manen, 1990).

The interview ended with an open concluding question: “Is there anything else you feel is important that we did not cover and you would like to tell me about?” In some cases, this question facilitated more examples and clarifications of material previously recounted. All interviews were conducted in the participants’ homes at a time convenient for them so that they would be in a familiar environment (Creswell, 2007), providing the conditions to freely and spontaneously develop their story in their own way, pace, and language. As compensation for their participation, each interviewee was awarded two coupons for coffee and pastry in a coffee shop of their choice.

### Data Analysis

The interview transcripts were analyzed by using a phenomenological approach in order to obtain a more profound understanding of the experience of meaningful aging from the participants’ perspectives. First, all interviews were read independently several times by four research assistants (who did not conduct the interviews), as well as by both authors, to obtain an overall impression of the participants’ experiences, until reaching a sense of immersion.

Next, the research assistants and the first author independently identified “meaning units” (Malterud, 2001) as expressed by the participants, using an “open coding” process (Strauss and Corbin, 1990). Such line-by-line coding (Charmaz, 2006) enabled identifying the manner in which each participant experiences the phenomenon under study by systematic queries, such as “Which processes and meanings are expressed in each line, sentence, and paragraph?” and “How, when, and why are they expressed?” These meaning units were then used to create descriptive categories of basic themes, thus constructing an initial framework for further analysis. The various categories and basic themes were then reexamined and compared for possible connections across individual meaning expressions as well as between participants. The final step, comprising a holistic examination of themes and their interrelations, was conducted to achieve a broad understanding of the participants’ experiences.

Although the data analysis is portrayed here as a linear procedure, the actual process was more cyclical, with each stage building upon its predecessor; each case was assessed discretely, as well as across all participants. As such, it involved a dynamic and repeated, back and forth, between the parts (i.e., texts and quotations) and the whole (i.e., the entire transcript), as well as between units of meaning and general themes (e.g., van Manen, 1990). This procedure enabled a close reading of the phenomenology of the participants’ experience, thus certifying confirmability. Furthermore, a fundamental methodological principle within the phenomenological framework concerns “bracketing” (Beech, 1999) in which the researchers set aside their experiences and attitudes, as far as possible, to adopt a fresh perspective on the phenomenon being explored (Moustakas, 1994). To address this, reflections and insights were summarized and then documented (van Manen, 1990). Furthermore, a triangulation of the interpretations was facilitated by an ongoing process of critical discussion of the data between the authors, as well as by an additional independent reader not involved in the previous stages. Finally, all interpretations were grounded in direct and rich excerpts from the interviews (Stiles, 1993).

## Findings

Three prominent themes emerged from the data analysis: (1) central challenges and concerns described by the participants as characterizing their experience at this life stage; (2) strategies employed by the participants to cope with concerns and to live a meaningful life in old age; and (3) resources and strengths that facilitate coping strategies and enable thriving for the participants. These themes will be presented along with illustrations derived from the participants’ interviews. All quotations provided by participants are vital and important for conveying the study’s findings and conclusions. Written informed consent to present this information was granted, and in order to protect their identities, participants’ names and gender are omitted and ages appear in ranges.

### Central Challenges and Concerns

When asked about their experience of current life stage, an essential existential concern of death and increased recognition of limited time, in various forms, was evident across all interviews. This was often articulated as a sense of life being too short and that there is a time pressure to achieve one’s personal hopes and desires, as can be seen in the following example:

*I really fear death. I find myself thinking a lot about it. An average person dies at the age of 70–74, and you don’t really have a chance to do anything… people around me tell me not to think about it, but I can’t help it. I want to achieve more things in life, to do more things, to see my granddaughter growing up. She is three years old; in nine more years she will have a bat-mitzvah. Will I live to see it?* [older adult, male, age 60–64].

The fear of death was portrayed directly as well as interwoven among other concerns raised by the participants, as reflected in the following two subthemes.

#### Fear of Losing Control

The acute acknowledgment of limited time ahead was experienced by the participants, not only as limiting the fulfillment of future possibilities, but also implying a loss of control. Loss of control was voiced as related to relinquishing responsibilities that had been central to their sense of personal identity, such as family roles. For example, a common concern related to the future of loved ones and ways to ensure they will continue to be taken care of and continue their lives without them. For example:

*The family becomes a central concern at this age. I mean, you have a limited time here, who knows when it [death] will occur, and you kind of fear, kind of worry… It’s like, I am married to my wife for the last 47 years, but I am also married to a constant anxiety that intensifies the older I get, regarding the welfare of my grandchildren, children… to make sure that everything will be alright* [older adult, male, age 70–74].

Loss of control was also voiced as reflecting decreasing personal independence—especially with regard to the participants’ physical condition—in terms of fear of deterioration and of physical pain and suffering, which is experienced as limiting the participants’ capacity to make use of the short time horizon ahead. For many participants having full, meaningful, and active life means to be independent, to choose freely how to live their remaining time. For example, in the following excerpt, this older adult recounted that lacking control of her physical condition goes hand in hand with the extent of her ability to fill her remaining time with valuable activities:

*Ever since I fell, I’m not free, I don’t go out, I don’t function. I used to cook and bake and do many things. Today, I can’t; my daughter does things instead… It’s like I sit and wait for my death, because when your physical condition deteriorates, it affects how independent you are and what you do with your time. You are not in control* [older adult, female, age 70–74].

Losing control over one’s activity and time was noted repeatedly, not only with regard to physical limitation, but also a mental one, as can be seen in the following example:

*I think that dealing with old age is also seeing its beautiful sides, living the time you have left... For me, I’m more bothered by the prospect of a deteriorating mental state, becoming someone who does not understand what goes on around her or disconnected to reality. This is much more troubling for me than a physical limitation, like being in a wheelchair or in need for special care at home.... As long as I’m in control of my life and as long as I feel that I am aware of what is going on, it’s enough for me to deal with whatever challenges this age brings with it, knowing that I may not have much time*. [older adult, female, age 70–74].

The participants described their attempts to maintain control over their lives as much as possible, such as eating well and stay physically and mentally active. Some even asserted that retaining control over one’s life means being in control over one’s death as well:

*In my fantasy, if the situation gets really bad, I hope I’ll have enough cognitive and physical capacity to do something… Very few people go to Switzerland or somewhere [else] to end their lives, to take their lives in their own hands… For me, there is no question about it. I don’t want to die, but if I’ll get to a point of suffering terribly and have the ability to stop it, I will… It goes hand in hand with the fear of losing control. I want to control my life. It makes me happy to know that I can choose and do it. If I won’t be able to choose anymore, I don’t want to continue. If I look forward to the future, I think that it’s my way of dealing with my own death. Maybe someday there will be an option for elderly people to choose their death day. It might seem today as not Jewish and unrealistic, but I don’t understand why an old dog can and an old person cannot… I mean, an old dog that suffers, can just get an injection, so why can’t we have that option too?* [older adult, female, age 70–74].

#### Fear of Squandering Spare Time and Missing Out

Another manifestation of existential death anxiety evident in the interviews was a “fear of missing out” (FOMO). Some participants talked about their need to do as much as possible, to learn as much as they can, to experience as much as possible, and to “devour the world,” as one participant noted, before their time runs out. For example:

*I don’t have enough hours in a day… seriously, I don’t have enough time… I don’t want to miss out on everything that is out there. I can’t manage to do everything I want to with all of my activities. I’m short of time… I don’t have any spare time, and it’s good. I don’t want to be bored; there is so much to do* [older adult, male, age 75–79].

Concerns of missing out and squandering the limited resource of time voiced by the participants highlighted the high value placed on keeping busy and active, and avoiding the vacuum of “dead hours” that may trigger existential reflections about life and remaining time. According to the participants’ accounts, remaining active and busy, confer a sense of being in control, staying relevant, significant, and useful. The following participant, for example, shared her experience that keeping every moment full, keeping busy all the time, and filling her days with activities keeps her from getting bored and having empty spare time:

*I look for things to keep me busy, whether it’s reading books, watching movies, attending classes on many topics, some sports… I feel that I need to; I don’t want to be drawn into doing nothing, going through my days with no purpose*... [older adult, female, age 65–69].

Some participants even articulated a complementary sense of social expectation to remain active rising with retirement, encouraged by organizations to volunteer, to attend lectures and group activities:

*When I retired, I felt like I’m going to “grab” the world, to finally do all of the things you couldn’t do before, due to life’s commitments to family and work. And society encourages that, with a wide range of volunteering options and activities offered to retired people… the atmosphere was like, ‘so what are you doing on your pension? How do you spend your time? What are you studying?’ Like you owe something to the world, like it was expected from you… So, I started doing it, going to lectures and activities and dancing and singing, filling my days with activities* [older adult, female, age 75–79].

Making every moment count gives the participants a sense of precious time used, as this excerpt suggests:

*It’s getting up in the morning with a plan, knowing that there is something for you to do, that you have a plan. It is very, very important to me that my day is completely full. It is important for me to be active, to meet friends, to go to lectures… I hate wasting time; I don’t like to feel that time is fading away. I am frightened of getting bored, because you see, the kids are grown up, the grandkids are grown up, and they don’t need me to babysit every day, so to fill life with meaningful content… you need planning and doing. I’m afraid to reach the point when it will end. But of course, it will have to end at some point...* [older adult, female, age 75–79].

Such a realization ignited a sense of urgency in some participants, reflected in crystallizing the things that are essential and significant to achieve in a limited time, which is also free from external obligations; as the following excerpt highlights:

*We always postpone things because we feel that we don’t have enough time to get to them... We think that we will reach a certain point in life when we will start doing the things we always wanted to do, but when you finally ‘have time,’ and I say that in quotation marks, you realize that it’s still not enough; you don’t have the time to do everything you thought of doing, and that’s frustrating. You keep promising yourself that ’now I can really start living because I finally don’t have all of these obligations to get up in the morning and run to work and send off the kids’... and then you realize how much you’ve postponed. Apparently, life is too short, and we don’t really know how much time we have left* [older adult, female, age 70–74].

The participants also acknowledged the dual consideration of not wasting time by filling it with meaningful daily activities and a life imbued with a sense of purpose, for example:

*I see that there are times when I’m not focused on a specific target, and then I waste my time not doing things that are meaningful for me. And there are things that are important to me, things that I really want to do, but due to a lack of thinking ahead or planning, I postpone them or don’t do them properly… It is important for me not to waste time, not only because I think that there is a limited time to each person, but because everyone has missions to fulfill in life, and it’s a pity to postpone them. It’s not just that we are born and then die* [older adult, male, age 60–64].

Other challenges involved with old age articulated by the participants included experiencing loneliness and concern that where essential family and work roles have been downgraded, they will be left “outside of the game,” as expressed by one participant. In other words, they fear becoming useless and unable to contribute to society, as this excerpt illustrates:

*When a person retires, he or she may feel that they are meaningless to the world. The most important thing for a person is to feel meaningful, that their existence is not meaningless. For many people retiring in old age, their main problem, I think, is that they feel insignificant, they feel useless and unneeded… when you have meaning, when life has content, then there is something to live for* [older adult, male, age 65–69].

Some participants referred to this “void” directly as potential danger characterizing old age; for example:

*Many people in their older years are not in the mood to do anything. I’m not in the mood sometimes, too… because we are tired, tired of life; and people say that we have done enough in life, and this is the time to rest. But what does it mean “to rest?” Do nothing? Does it mean that “our time” is over, and now we should live an empty life? Unfortunately, this may cause depression, and many people my age are depressed* [older adult, female, age 70–74].

Other participants referred to loneliness as a given as part of their old age:

*The most difficult thing to endure at my age is, for example, loneliness… I feel the loneliness in the afternoons and in the evenings, because in the mornings, I am very active… But I find some comfort in knowing that there are other people who share these feelings* [older adult, female, age 75–79].

*This is one of the problems of people my age; they don’t leave their homes, and they are lonely and miserable. When someone is lonely, he is miserable. For religious people, it may be less of a problem because they meet regularly at their house of worship, whether it be a synagogue or a church… an observant Jew goes to the synagogue… A place where people come together. There is togetherness, and when there is togetherness, life is better* [older adult, male, age 80–84].

### Strategies for Coping With Challenges in Old Age

Although the interviewees were not asked directly about coping strategies, they described various strategies they employ in order to deal with the concerns raised and to live a more meaningful life at this life stage. These include two overarching recurrent categories: (1) establishing active routine; and (2) contribution to others and the world.

#### Active Routine

Many participants discussed the importance of establishing an active schedule or routine, particularly given the blocks of discretionary time available after retirement and no longer having child rearing in their purview. According to their accounts, an organized schedule of activities provides a sense of order and purpose—having something to wake up for in the morning:

*You need a daily routine with at least one activity a day. Every day, I have something else to do; I try to divide my days so that every day, I’ll have something different to do, that there won’t be a situation where I have nothing to do* [older adult, female, age 75–79].

Acknowledging that maintaining an active lifestyle encourages development and growth, even in the face of life’s challenges and adversities, is also evident in this excerpt:

*Even though my son passed away, I kept my cheerful mood. I don’t ask what life brings to me. I try to keep going, to keep doing things. And that is also true for dealing with the challenges that age brings with it. If you continue to learn, improve, grow, you are motivated to live. If I’d reach a point where I won’t have an interest in life, that will be the day I’ll die. Then I will have no point in living, nothing to aspire to* [older adult, male, age 60–64].

The participants asserted that an active lifestyle is a buffer, not only for boredom and existential reflections on limited time horizon, but also as an antidote for withdrawal from life, and even for depression, for example:

*When I retired, I knew that if I’d sit at home, I’d decline quickly. I would lose interest. So, I started drawing; I have loved drawing all my life, but I stopped for a long time because there was always something to get done. Now, I returned to it. I said to myself, “this is my time.” I also volunteer with elderly people and with young single parents… I got on with my life, and I didn’t fall into depression like I feared I would. I had reasons not to.* [older adult, female, age 75–79].

#### Contribution to Others

Another common strategy expressed by the participants was pursuing activities in which they can help, care, and contribute to others, whether in close relationships and\or through volunteering in various venues. Participants spoke about their desire and commitment to help others, as can be seen in the following example:

*At my age, I realize that my time here is limited, so I try to do good. The most meaningful thing is volunteering… It makes you feel that you can contribute. You give to someone and you immediately receive something in return… This is how it is with an elderly woman in my neighborhood, for example. I go there to keep her company so she won’t be alone and bored, and she thanks me so much, I tell her that I enjoy coming to her, that she doesn’t need to thank me so much... At times when I can’t visit, I feel really bad, and when I do [visit], it is fulfilling for me...* [older adult, female, age 75–79].

The following participant, too, referred to helping others through volunteering as a strategy she employs which enables her both to cope with and to find meaning in face of challenges at this life stage:

*I volunteer a lot; I volunteer in hospitals, I am an ambulance driver… My days are more satisfying and fuller today than when I was working… It gives a lot, helps you deal with life’s complexities. And it starts with being human, being kind to others. As simple as that. It’s like plants that you give them water. What did you do? You just give them water, it’s not a big effort. But what it does to the plant... wow, it makes it bloom, it makes it happy. I do good for other people, and it makes me feel good, too; it’s very satisfying for me. When you approach a sick person on the ward—and I volunteer in the oncological ward—it’s very difficult; he [the patient] has no one, and you bring him a glass of water, and he thanks you so much as if you did something huge, and it’s gratifying that you succeeded in causing him to smile… this person who was in very serious condition. At this age, you realize it’s not just about you, personally. I don’t think only about myself. It makes your life meaningful; this is a reason to get up in the morning, isn’t it?* [older adult, female, age 60–64].

### Resources and Personal Strengths as Facilitators for Coping and Meaningful Aging

Several resources and personal strengths emerged from the interviews, as supporting the strategies the participants used to cope with the challenges and concerns they voiced and to living more fully. These included three central categories: (1) connection and belonging; (2) openness and savoring experiences; and (3) positive attitude and moderation.

#### Connection and Belonging

The participants articulated the important resource of being a part of something bigger than themselves. This manifests itself through maintaining close relationships with family and friends, attending study groups, congregation, and community involvement, to a larger scale of belonging to a nation or humanity. For example:

*Meetings in the company of friends, being a part of a big group of like-minded people, all of that makes me feel meaningful and relevant; they notice if I’m absent. We are a group of friends from work, friends for the past 50 years… I think that these meetings contribute to my mental health, they help me open up and share my experiences and challenges, even concerns. I feel I’m not withdrawing or secluded, I belong* [older adult, male, age 75–79].

Being part of a community appears to provide participants with a sense of having a secure anchor in the midst of the unique challenges they face at this life stage:

*Community is a real anchor for me… I find myself making considerable efforts to maintain it. As people pass away, the community becomes smaller, and I hope it won’t dissolve… because it’s a real anchor for me, something I can rely on…We meet, and people ask me how I am and take an interest in me. I can feel that they care. You know, there’s a saying: “It is better to have a close neighbor than a distant brother”... Community is that someone who can support me daily*… [older adult, female, age 70–74].

The participants referred to being a part of religious community as factor which enables to negate age-related concerns, for example:

*We are part of a community, the synagogue, the friends around, it’s very important. It is very important for our daily lives, especially now, at this age. I’m a big believer in community life; this is why I devote time to attending the synagogue, to meeting friends, engaging in activities for the benefit of my neighborhood and community. It takes up time, but it fills you with meaning; it’s important for me… I’m filling my time in a meaningful way… It’s knowing that I’m making meaningful use of my time and that I continue to contribute to society. I mean, I’m a part of something, it gives you a sense of being whole. I’m part of a family, part of society… I think this is the secret of well-being in old age; it can help you cope with all of the storms and turbulence of old age. I see people in different places, people that have nothing, attached to nothing… What kind of life is that? You wake up in the morning, read some newspaper, watch some television, eat, and go to sleep. What kind of meaning does such a life have?* [older adult, male, age 70–74].

Some participants even referred to a broader community, such as a nation:

*To be part of a bigger group enables you to deal better with things. This is what gives meaning to our lives… We are not loners that live merely to survive; we live because we are part of society. This is what holds us, this is what I think gives life purpose and meaning… In the broadest sense, the State of Israel and the Jewish people, it’s a community, a family. Otherwise, what are we doing here? I could live a great life in the USA… But we are here because there is a meaning to the fact that we live here in this society, in Israel*… [older adult, male, age 75–79].

Other than belonging to a community, close relationships and making new connections were also discussed as important resources for coping with existential concerns, such as loneliness. For example:

*I like connecting with people… when I sit on the bus, I try to sit in the back and seek out someone to talk to… Being in touch with friends, with people, makes you feel less alone, I call them and ask how they are doing… It is important for me to keep in touch with people I know. It’s gratifying for me, fills my days with content* [older adult, male, age 80–84].

Whether secular or religious, having a sense of connection and belonging also involved a broader spiritual experience of being a part of something bigger than themselves, that they have a role in the great scheme of things, and that their lives have purpose. For example:

*Knowing that you are part of something greater than yourself, that you have an affinity with God’s creation and eternality, is very comforting. You are not just an ant in infinity, dust in the wind… there is some sort of big plan that we all take part in, you have a significant part of it. That’s very comforting* [older adult, male, age 65–69].

#### Openness and Savoring Experiences and Encounters

The participants also described the importance of being open to the world to experience and to learn new things as a source from which they can derive strength, expand their horizons, and develop further:

*I continue learning and developing. It keeps me fresh and young… this is the thing that keeps me going… There is the Sisyphean work at home, which is cleaning and cooking… this seem totally Sisyphean to me. So, my priority is learning; this is something that makes living worthwhile, especially at my age* [older adult, female, age 70–74].

The personal strengths of love of learning and curiosity described by the participants as a resource enabling them to cope and experience life more fully was also prominent in their desire to know as much as possible about a topic that they are interested in, to visit new places, to experience more, for example:

*Curiosity gives me patience… there are things that I don’t understand yet and I’m open to learn more about. It’s a quality which is also important to life in general, I think, looking at problems from different angles instead of thinking you already know the solution and denouncing the new perspective* [older adult, male, age 60–64].

Being open to the world as a resource also involved the importance of savoring life’s gifts such as nature:

*When you experience your surroundings, you gain peace of mind… I have a neighbor whose husband passed away a month ago, and I told her to “take your life into your own hands,” don’t let yourself sink... Sinking is easy… go out to the garden and watch the plants, feel them, water them; it gives you a lot to go out to the garden… plants are grateful, they know how to thank you, they flourish and bear fruit… plants are healing, the green, the colors, the bloom. You see it flourish. If you don’t water it, it looks impoverished and shriveled, and just a little water and a day later, it suddenly blooms. It’s a wonderful thing, there is so much to learn from plants*... [older adult, female, age 75–79].

Such appreciating for beauty, noticing beautiful things that others may overlook, was experienced as an attitude, allowing the participants to frame routine errands not as a waste of time, but as an opportunity to enjoy the beauty the world has to offer, as “the fuel for the rest of the time” (older adult, age 70–74). For example:

*When I used to wait for a bus, my children used to tell me sometimes to take the car, but I insisted [on taking the bus]. I would answer ‘wait a minute, see all the beauty you can see when you have time to look out the window, the many beautiful flowers I see when I walk, so many things one can enjoy. Don’t see it only as the difficulty of boarding the bus, there are so many beautiful things out there, too* [older adult, female, age 70–74].

#### Optimistic Attitude and Moderation

Another common resource highlighted by the interviewees was a positive and moderated perspective on life. This involved taking responsibility over one’s attitude and perspective toward life and actively deciding how to deal with various life situations and endure unavoidable suffering, an intrinsic part of life, particularly in old age. For example, This participant pointed out the importance of one’s responsibility to appreciate life rather than complaining:

*I’m giving this advice to anyone who is aging: ‘take your life in your own hands and enjoy it. Don’t complain.’ We may have challenges, but when we complain, we only see the problems; focusing on what makes life wonderful makes you more resilient*… *As long as I’m alive and wake up in the morning, it’s a lot* [older adult, female, age 60–64].

The participants described positive perspective as enabling coping with life’s adversities and the challenges that age brings with it:

*We’ve had a great deal of tragedy in our family… I don’t cry about my misfortune, and many times. I say that I cannot change it, so I try to make good use of what I have… It’s a philosophy of life. I still love life. I am happy with life, and I try to see the good in it. What will I get from seeing what is bad? I think that this is a healthy way to live, not to let life be wasted* [older adult, female, age 70–74].

Participants articulated a more pragmatic, balanced, and moderated view of life that has become stronger with age. In the face of concerns and challenges, they have learned to accept things, to be satisfied with what they have, and to focus on what matters and is meaningful. As this excerpt exemplifies:

*Our whole life is like a movie. At this age, we start remembering things we didn’t have time to think about, to reflect… There are things we’ve done that make us happy, others that make us angry at ourselves, satisfied or disappointed, you analyze your life from different angles looking back. However, one of the gifts of old age is the serenity that comes with it. This is why they say elderly people have life wisdom… life has taught them* [older adult, female, age 70–74].

Participants attested to the fact that their views and attitudes have softened in order to deal with the limited time ahead and with other concerns they face. They feel less strongly about things that triggered emotional responses in the past; now they are more willing to move forward and leave the struggles behind:

*I don’t hold grudges anymore. When I was young, if you have a very good friend, and she suddenly disappointed you because she did this or that, you really feel like being angry with her… but the more I age, I realize that life is beyond all of these minor issues… You need to be wise, and I feel that I’m wiser, I don’t hold grudges anymore, it’s a waste of time. I feel like a winner, that I can move on and not get stuck on things that don’t really matter…* [older adult, female, age 65–69].

*I used to fight for justice. Today, much less. It’s important for me to be with my family, to celebrate the good things in life, not fight over who’s right… I try not to take [things] to heart… I try to change whatever I can, and the things I can’t, I choose not to take to heart like I used to*... [older adult, female, age 60–64].

Many of the participants have come to accept situations that they cannot change, such as medical conditions, limitations that come with age, and losses they have experienced. They choose to concentrate on what seems to be most important for them:

*There is an Indian saying I like that says that whatever you can change -change, and what you cannot, live with it. Don’t be stubborn and hit your head against the wall. I’m not stubborn. I know my limitations, both physically and financially, and also, of course, age limitations, and today I don’t really want to change things as I used to when I was young. I want to be a part of reality and life as much as possible and to flow as much as possible. It is what it is* [older adult, male, age 65–69].

*Maybe it’s universal, that people moderate with age. I feel that I have experienced general moderation in all of my view of things, of life. It’s more like wisdom, cautious decision making, less impulsivity. I don’t feel an obligation to accomplish things, to ‘show off.’ It’s more about living peacefully* [older adult, male, age 60–64].

These also highlight the capability to pause to be grateful for the good things in life, rather than taking them for granted. For example:

*We live from day to day. We don’t know what tomorrow will bring. It helps to be grateful, to see what we have and thank God… What we have is sufficient… There are so many good things in our lives, but we need to stop and recognize them* [older adult, male, age 80–84].

Wisdom and moderation were supported by having a way of looking at the world that makes sense, feeling that their life experience has made them wiser. They have accumulated more points of view and are better able to look at the bigger picture. For example:

*Sometimes, I see from the side people who are dealing with nonsense, arguing and fighting over nothing… “Look around you, see the world. You are fighting over nothing. No one promised us a rose garden.” There are things in life which are not so pleasant, but you learn to see it in perspective and not blow up the world over it* [older adult, female, age 70–74].

In sum, the findings suggested three overarching themes, which frame the essence of the participants’ lived experience in a holistic way. The first theme offers an insider’s perspective of the major concerns and challenges that aging raises, according to the interviewees’ own experience. The second theme offers practical strategies employed by the participants to cope with such concerns, and the third theme illuminates potential resources which facilitate coping strategies with the above concerns.

## Discussion

The purpose of this qualitative study was to explore older adults’ own perspectives of their aging experience, their perceived challenges and resources that enable them to cope with such challenges in their day-to-day living. Interest in aging well has increased in recent years, as conceptualized through various theoretical frameworks, such as healthy aging, positive aging, active aging, and successful aging (e.g., Poon and Cohen-Mansfield, 2011; Foster and Walker, 2014; Martin et al., 2015). However, despite mounting knowledge about the factors contributing to wellness in old adulthood (e.g., Vaillant, 2002; Ryff et al., 2016), evidence for a greater presence of meaning among older adults (Steger et al., 2009), which may serve as a protective factor (e.g., Reker, 1997) in later life, only few studies have focused on these issues from older adults’ own perspective. This lacuna was corroborated in a recent review of hedonic and eudaimonic well-being in old age through the lens of positive psychology (Araújo et al., 2017), revealing that while 29 quantitative studies have been conducted on this topic in the last decade, only four of them were of a qualitative nature. The authors called for a greater use of qualitative studies with older adult samples, as interviews and open questions regarding well-being may lead to more productive data collection (Araújo et al., 2017). Thus, an open-ended approach to exploring older individuals’ experiences may lead to a more comprehensive and in-depth understanding of the theoretical and practical implications for this age group, as it takes into account the challenges and concerns they are faced with, as well as the resources and coping strategies that they develop.

The present study illuminates several integrative contributions, expanding existing knowledge. The findings suggest that the essence of the experience of Israeli older adults reflect an amalgam of both existential concerns and personal resources, particularly character strengths, which facilitate coping with challenges and concerns as well as contribute to living more fully at this life stage.

The participants in the present study articulated a major concern of death, both directly and indirectly by the concern of limited time, fear of losing control and the concern of squandering time and missing out. This major concern corresponds to Yalom’s (1980) consideration of death as the most prominent of the four existential concerns. Among older adults in particular, the awareness of personal death may trigger psychological pain and distress. According to terror management theory (TMT), developed by Ernest Becker (1973), the motivation to deny death is viewed as a unifying concept for human behavior. Being human involves the unique paradox of the self-preservation instinct concurrent with self-consciousness: humans are aware of their own vulnerability and mortality, and at the same time, are motivated to avoid the terror of death. This can be accomplished through granting the world meaning and value that go beyond death and vulnerability by investing in a cultural worldview and securing self-esteem (see Martens et al., 2004). The concern of death was accompanied by the participants’ experience of being left “outside the game” and feeling useless, where previously critical roles in the family and work domains have been considerably modified. This notion is in line with the importance of remaining useful, as described in narratives in other studies (e.g. Lieblich, 2014). This echoes Freud’s famous response to the question regarding the core components of psychological health and meaningful life: “Love and work are the cornerstones of our humanness” (see Elms, 2001). These two dimensions are particularly evident in old age, with theories in social gerontology showing the centrality and linkage between these two dimensions (Zaidi and Howse, 2017).

Disengagement theory, one of the first theories of aging, postulates a view of old age as a time of life when people step back from various commitments and social roles. In response to disengagement theory and in line with the frameworks developed to incorporate other more positive experiences of life in older age, such as successful aging, positive aging, and productive aging, in line with previous research (Lieblich, 2014), the findings of the present study highlight the desire of older adults to remain active participants in society through creating opportunities for social connectedness, contribution, and belongingness. This is in line with the strength and vulnerability integration theory (e.g., Charles, 2010), suggesting that a limited time horizon alongside accumulated life experience may enable older adults to develop strategies to adjust their emotions and organize their surroundings to avoid or minimize negative experiences. One of the contributions of the present findings is the importance of active lifestyle, as a facilitator of a sense of coherence, order and consistency, in the experience of older adults. The participants’ volitional attempts to structure their environment by establishing an active routine, despite of the loss of previous roles, appear to provide them with a sense of control and meaning. In this sense, “bottom-up” real-life findings support previous empirical laboratory studies, suggesting that engaging in routinized behaviors may enable a connection to a larger context and cultivates a sense of meaning (e.g., Heintzelman and King, 2019). Routines allow for structure (Heintzelman et al., 2013) as well as coherence and comprehensibility—the extent to which life makes sense and characterized by consistent connections (e.g., Baumeister, 1991; Baumeister and Vohs, 2002). Routines have also been mentioned as contributors to positive aging (Lieblich, 2014).

Another important contribution integrates classic theoretical models of meaning in life with the actual experience of older adults. The strategies and resources voiced by the participants correspond with Frankl’s (1959) pathways to discovering meaning in life: the creative, experiential, and attitudinal. *The creative pathway* emphasizes what one gives to the world, in terms of creation (e.g., volunteering, deeds, dedication to personal goals). This pathway incorporates using individual assets to contribute to the society more broadly. *The experiential pathway* emphasizes what one takes from the world, in terms of experiences and encounters (e.g., nature, art, humor, roles, love, relationships). *The attitudinal pathway* emphasizes the stand or attitude one takes regarding situations that cannot be changed or unavoidable suffering. Through the implementation of a positive attitude in the face of aversive or traumatic events, Frankl believed that the individual can find increased meaning (Frankl, 1959; Batthyany and Russo-Netzer, 2014). Closely related is the socioemotional selectivity theory (Carstensen et al., 1999), which suggests that, as our perspective of time changes with age, our motivations shift accordingly. Indeed, the participants in the present study voiced an acknowledgement of concerns and challenges that characterize this life stage but at the same time to leverage their concerns to take responsibility over their lives and create new structures to contribute to society through volunteering, dedication to a community, and close relationships. These findings are consistent with previous research indicating that successful aging requires a balance between self-acceptance/self-contentment and engagement with life and self-growth, within it representing themes of realistic self-appraisal, a review of one’s life, focusing on the present, engaging in novel pursuits, giving to others, engaging in social interactions, maintaining routine, engaging in exercise, and having a positive attitude (Reichstadt et al., 2010; Lieblich, 2014). This essence is also central to Erikson’s theory of psychosocial development, where the task of the adulthood stage is the resolution of the generativity versus stagnation crisis, reflecting a “need to be needed” (Erikson, 1968, p. 138), contributing to future generations by productive and creative endeavors. Overall, Frankl’s (1959) three pathways reflect self-transcendence: toward others, through volunteering or relationships; toward the world, through experience or creation; and beyond given conditions and circumstances. In a similar vein, the participants in the present study articulated their capability to transcend beyond individual self and daily hassles, to the great scheme of things that “truly matter,” balancing accepting the past, living more fully in the present, and striving to make a contribution and leave a legacy for the future, through their attitudes and actions.

Utilizing a “bottom-up” perspective, we also found that the participants articulated personal strengths as resources and channels to both cope and move toward a more full and meaningful life, in their experience. Interestingly, most (but not all) of the strengths that emerge from the interviews correspond to several character strengths identified by the *VIA* classification (Peterson and Seligman, 2004). More specifically, the participants’ active strategies of building daily routines and contributing to others, demonstrated through experiencing and creating, can be seen as facilitated by character strengths which belong to the humanity cluster (i.e., kindness and love) and to the wisdom and knowledge cluster (i.e., curiosity, love of learning, appreciation of beauty). The attitudinal strategies of positive and moderated attitude can be seen as facilitated mostly by strengths comprising the transcendence cluster of character strengths (i.e., hope, gratitude, spirituality). Positive spirituality in aging was defined by Crowther et al. (2002) as an internalized relationship with the sacred and transcendent that is not bound by race, ethnicity, or class, and as an important contributor to the wellness of the self and others. Indeed, studies have shown that spirituality tends to increase in later adulthood (e.g., Moberg, 2005; Koenig, 2006), and it has been considered recently as an important ingredient of well-being in positive psychological studies of old age (e.g., Sadler and Biggs, 2006; Cowlishaw et al., 2013).

The participants also voiced an attitudinal approach to a life of moderation and balance that emerged as standing on its own, rather than as reflective of specific character strengths that appear in the *VIA* cluster of temperance (prudence, forgiveness, humility, and self-regulation). Such general moderation was voiced as an experience of maturation and life wisdom, which enabled them to look at the bigger picture through a more pragmatic and balanced view of life. Facing concerns and challenges, they learned to accept things, to be satisfied with what they have, and to focus on what matters and is meaningful. The acceptance aspect of wisdom is in line with previous findings (Reichstadt et al., 2010). Lieblich (2014) discusses moderation and wisdom as a balance between silence and preaching, distance and closeness and control and withdrawal. This is also in line with developmental theories, such as those of Jung (1959) and Erikson (1968), which emphasize maturation, increased introspection, and resolution. Future studies may consider moderation as a character strength that may reflect a distinguishing developmental characteristic of old age and stand on its own.

Overall, the present study extends previous findings by offering a potential ‘bridge’ between existential concerns and positive psychological resources and character strengths, through the perspective and voices of elderly individuals. Our findings also suggest practical implications for policymakers and caregivers to consider interventions with the elderly that facilitate the balance between the acknowledgment and acceptance of existential concerns, while creating a safe space for self-transcendence, through creative, experiential, and attitudinal pathways of active and relational engagement by using their personal character strengths. This is also in line with calls to integrate spiritual issues into the psychological care of older adults (e.g., Ortiz and Langer, 2002). The findings and gained insights from the present study reflect the importance of qualitative research methods in capturing the richness and complexity of a phenomena, in-line with previous suggestions regarding this approach’s focus on understanding how individuals interpret or ascribe meaning to a given experience (e.g. Hodge, 2001). Listening rather than measuring has the potential of revealing “fresh categories of meaning that quantitative studies may not have discovered” (Blieszner and Ramsey, 2002, p. 36). In the context of older age, quantitative assessments follow stringent inclusion criteria leading to omission of participants and data, and largely risking neglecting the subjective component of successful aging (Von Faber et al., 2001).

### Research Limitations and Directions for Future Research

Given that the present study was conducted within the specific sociocultural context, an exploration of other cultures would constitute an important direction for future research. Such exploration may suggest cross-cultural as well as individual differences in the manner in which people experience, articulate, and discern their existential concerns, coping strategies, and experiences of aging. Furthermore, all participants were from a Western, moderately individualized culture. More specifically, the Israeli context where the study took place may have additional unique characteristics that underscore the potential salience, significance, and importance of searching for meaning in life. An exploration of the issues explored in the present study among different populations may illuminate different concerns that are common to the aging population, such as existential freedom, whereas other concerns may not be as prevalent, such as the concern of death or existential vacuum.

Moreover, the recruitment strategies employed in the current study were designed to recruit active older adults, in order to explore their own perspectives regarding the day-to-day living experience and perceived resources at this life stage. Thus, despite their contribution, the current findings should be interpreted with caution and cannot be generalized to other contexts or populations. An important challenge for future research might be to examine how the themes identified in the present study might apply in other older adult subgroups that are less active. Also, this study adopted the WHO (World Health Organization) (2002) and the United Nations standard of age 60 to describe “older” people. However, there may be individual differences within this group (e.g.,60-year-olds and 80-year-olds), as there are many countries where 60-year-old adults are still active and continue to work in the formal labor market and contribute through informal work and voluntary activities. Hence, an important direction to future studies may be exploring individual differences in the experiences between younger older adults and older adults, which may also help to shed further light on this phenomenon.

Regarding the *VIA* model of character strengths, this study raises questions that should be explored in future quantitative and qualitative studies, such as the role of character strengths in coping with existential concerns and enhancing meaning along the lifespan, and the place of *moderation* as a potential new character strength relevant for meaningful and authentic aging.

Qualitative research methods in general and phenomenological research in particular do not strive for representation, generalization, or the extraction of an objective truth from the findings (Patton, 2002). Instead, they strive to gain an understanding of the processes by which human beings construct meaning from their experience (van Manen, 1990; McPhail, 1995) and allow for the prospect of extrapolating the findings to other settings or contexts by providing rich data suggesting ideas that merit further exploration (Elo et al., 2014). Thus, altogether, the qualitative-phenomenological nature of the present study has facilitated an exploration of the participants’ experience “bottom-up” in more depth and illuminated phenomena that correspond to previous theory and studies, and also suggest directions for future quantitative studies.

## Data Availability Statement

The datasets generated for this study are available on request to the corresponding author.

## Ethics Statement

The studies involving human participants were reviewed and approved by Department of Counseling and Human Development, University of Haifa. The patients/participants provided their written informed consent to participate in this study.

## Author Contributions

PR-N and HL-O have made substantial contributions to the conception of the study, the acquisition, analysis, and interpretation of the research data, and in preparing the manuscript for publication.

### Conflict of Interest

The authors declare that the research was conducted in the absence of any commercial or financial relationships that could be construed as a potential conflict of interest.

The reviewer JD declared a shared affiliation, with no collaboration, with one of the authors, PR-N, to the handling editor at time of review.
